# A novel strategy of radiofrequency hyperthermia (neothermia) in combination with preoperative chemoradiotherapy for the treatment of advanced rectal cancer: a pilot study

**DOI:** 10.1002/cam4.431

**Published:** 2015-02-09

**Authors:** Hisanori Shoji, Masahiko Motegi, Kiyotaka Osawa, Noriyuki Okonogi, Atsushi Okazaki, Yoshitaka Andou, Takayuki Asao, Hiroyuki Kuwano, Takeo Takahashi, Kyoji Ogoshi

**Affiliations:** 1 Division of Surgery, Hidaka HospitalGunma, Japan; 2 Division of Radiology, Hidaka HospitalGunma, Japan; 3 Department of Oncology Clinical Development, Graduate School of Medicine, Gunma UniversityGunma, Japan; 4 Department of General Surgical Science, Graduate School of Medicine, Gunma UniversityGunma, Japan; 5 Department of Radiation Oncology, Saitama Medical Center, Saitama Medical UniversitySaitama, Japan; 6 Division of Cancer Diagnosis and Cancer Treatment, Hidaka HospitalGunma, Japan

**Keywords:** 8 MHz radiofrequency capacitive heating device, capecitabine, hyperthermia, intensity-modulated radiotherapy, rectal cancer, reference point

## Abstract

The safety of weekly regional hyperthermia performed with 8 MHz radiofrequency (RF) capacitive heating equipment has been established in rectal cancer. We aimed to standardize hyperthermia treatment for scientific evaluation and for assessing local tumor response to RF hyperthermia in rectal cancer. Forty-nine patients diagnosed with rectal adenocarcinoma were included in the study. All patients received chemoradiation with intensity-modulated radiation therapy 5 days/week (dose, 50 Gy/25 times) concomitant with 5 days/week for five times of capecitabine (1700 mg/m^2^ per day) and once a week for five times of 50 min irradiations by an 8 MHz RF capacitive heating device. Thirty-three patients underwent surgery 8 weeks after treatment. Three patients did not undergo surgery because of progressive disease (PD) and 13 refused. Eight (16.3%) patients had a pathological complete response (ypCR) after surgery. Among patients without surgery, 3 (6.1%) had clinical complete response (CR) and 3 (6.1%) had local CR but distant PD (CRPD). Ninety percent of ypCR + CR patients were shown in 6.21 W min^−1^ m^−2^/treatment or higher group of average total accumulated irradiation output with 429°C min^−1^ m^−2^ or higher group of total accumulated thermal output. However, a patient with CRPD was in the higher total accumulated thermal output group. We propose a new quantitative parameter for the hyperthermia and demonstrated that patients can benefit from mild irradiation with mild temperature. Using these parameters, the exact output, optimal thermal treatment, and contraindications or indications of this modality could be determined in a multi-institutional, future study.

## Introduction

In rectal cancer, the higher recurrence rate, especially the higher local recurrence rate after surgery compared to colon cancer, is a major problem. Since the National Comprehensive Cancer Network Practice Guidelines for treatment of stage II and III primary rectal cancer were specified in 2009, neoadjuvant chemoradiotherapy (CRT) has become the standard treatment for locally advanced cancer worldwide, except in Japan. Many studies have demonstrated that neoadjuvant CRT increases local control, but has no effect on overall survival 1,2.

Bosset et al. recently reported that adjuvant fluorouracil-based chemotherapy after preoperative radiotherapy (with or without chemotherapy) does not affect disease-free survival or overall survival 3. New treatment strategies incorporating neoadjuvant therapy are required for rectal cancer.

Although hyperthermia has a long history as an oncological treatment and is widely used in clinical practice in several medical fields, unfortunately, this treatment modality has not been accepted widely in cancer therapy, because of an absence of strong scientific proof and stable, reproducible treatment quality. Since multicenter studies were difficult to perform, the clinical effect of hyperthermia was questioned 4,5. One of the reasons for these problems is the lack of standardized parameters for evaluation of the efficacy of this modality, that is, there is no reference point for this therapy. Consequently, interdisciplinary scientific analyses cannot be performed. In general, hyperthermia performed over five times was better in combination with chemotherapy, with or without radiation therapy than each modality or surgery alone. However, there was no indication whether each hyperthermic treatment was of the same quality. Moreover, troublesome problems such as the “hot-spot phenomenon” (specific acute to subacute side effects caused by the electrical interface) are reported to be present in up to 60% of cases, and cause pain during radiofrequency (RF) hyperthermia treatment. As a result, treatment cannot be continued without lowering the output 6.

We reported previously that in locally advanced rectal cancer, 5-fluorouracil-based CRT concomitant with RF hyperthermia can be performed safely and can improve the pathological complete response (ypCR) rate as well as tumor downstaging 7,8.

The aims of this study, which was from the perspective of thermotherapy, were to define the quantitative parameters for standardized treatment and to evaluate the effects of RF hyperthermia device condition on local tumor control in rectal cancer patients who were treated with preoperative hyperthermochemoradiation therapy (HCRT).

## Materials and Methods

Forty-nine consecutive patients diagnosed with rectal adenocarcinoma between December 2011 and January 2014 were included in this study. All patients received pre- and posttreatment diagnostic examinations at Hidaka Hospital. Staging for distant metastases was determined with computed tomography (CT) or positron emission tomography/computed tomography (PET/CT) of the abdomen and thorax, while tumor and node stage was classified mainly by magnetic resonance imaging (MRI). The extent and location of the tumor were classified according to tumor node metastasis (TNM) staging 9. All patients underwent preoperative HCRT at Hidaka Hospital. CRT consisted of intensity-modulated radiotherapy (IMRT) five times weekly at a dose of 50 Gy/25 times and oral administration of capecitabine 1700 mg m^2^ per day, 5 days/week for five times. Five thermotherapies were performed once a week with 8 MHz RF capacitive heating equipment (Thermotron RF-8; Yamamoto Vinita Co., Ltd., Japan). The study was approved by the ethics committee of the Hidaka Hospital and Gunma University, and each patient gave written informed consent.

### Chemoradiotherapy

IMRT was administered conventionally once daily five times/week using TomoTherapy® (Hi-Art® Treatment System ACCURAY®, Accuray, Sunnyvale, CA, USA). Radiotherapy consisted of 50 Gy delivered to the posterior pelvis in 25 fractions of 2 Gy. The planning target volume included the clinical target volume plus a 2.5 cm caudal margin and a 1.5 cm ventrodorsal margin. Concurrent preoperative chemotherapy was delivered in 5-day courses during the first to fifth weeks of radiotherapy. Capecitabine was administered orally at a dose of 1700 mg/m^2^ of body surface area per day.

### Hyperthermia

Hyperthermia was administered after radiation. RF hyperthermia using an 8 MHz RF capacitive heating device was applied five times for 5 weeks with 50 min irradiation. From December 2011 to November 2012, 19 patients retrospectively received hyperthermic therapy by Thermotron RF-8. The output was increased until complications such as pain occurred, following which output was decreased and subsequently, increased when pain subsided. Consequently, the output varied patient by patient. This no-rule method of irradiation was not standard. From November 2012 to January 2014, 30 patients prospectively received standardized increasing output (we called this neothermia) based on preceding data, which were dependent on patients’ characteristics before treatment, such as body mass index, thickness of the fat of the abdominal wall and internal organs fat area, total fat area, subcutaneous fat area, etc. Fat thickness of the abdominal wall and internal organs fat area, total fat area, and subcutaneous fat area were evaluated using CT. From retrospective data about complications, we had noticed that patients with complication showed bigger thickness of the abdominal wall, internal organs fat area, and total fat area than those without complications. Therefore, we classified patients into two groups as follows: (A) patients with <16-mm thickness of the abdominal wall fat, 100 cm^2^ internal organ fat area, and 190 cm^2^ total fat area, and (B) patients with any one of the factors previously described. For patients in group A, the output increase was 50 W/min, while in those in group B, it was 50 W/2 min. The operator started from 200 W and increased the output until complications occurred and then decreased the output by 100 W. Most patients did not complain and continued the first irradiation treatment. Subtracting 100 W output was judged as the optimal output dose without complications. From the second to fifth irradiation treatment, this output was applied for 50 min. We think these our methods were the first time in hyperthermia community. First of all, by using the quartiles of frequencies procedure by SPSS (IBM, Armonk, NY, USA), we simply applied to the classification of patients into four groups according to the average total accumulated irradiation output (TAIO; average W min^−1^ m^−2^/treatment). These were average TAIO of 6.2 or lower, 6.21–8.4, 8.41–10.43, and 10.44 or higher. Based on total accumulated thermal output (TATO; °C min^−1^ m^−2^) patients were classified into four groups as follows: 428 or lower, 429–466, 467–548, 549, or higher. Finally, we concluded to be enough to be two to three groups of TAIO and TATO in clinical setting.

**Figure 1:**
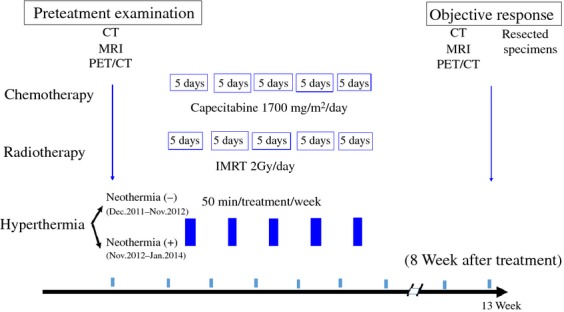
Protocol of this study. CT, computed tomography; MRI, magnetic resonance imaging; PET/CT, positron emission tomography/computed tomography; IMRT, intensity-modulated radiation therapy.

A sensor catheter with four temperature points was placed in the rectum in 12 patients while it was attached to the skin on the lateral abdominal side in 30 patients who received standardized therapy and in seven who did not. The accumulated surface skin thermal output of four temperature points was calculated from the estimated internal temperature of patients during the 50 min of each irradiation. An increased thermometry scale of the skin was added to the pretreatment axillar temperature of the patients to obtain a hypothetical internal body temperature that may be the possible core temperature. TATO was considered the heat effect of each treatment. Both temperature and output curves were recorded at 1-min intervals from 1 to 50 min. Body surface area was calculated by the DuBois formula (BSA = W^0.425^ × H^0.725^ × 0.007184) 10. Figure 1 summarizes the protocol of this study. All patients received the same CRT and hyperthermic therapy with or without neothermia.

### Evaluation of objective response

Each resected specimen was examined for histological changes after HCRT according to the histological criteria of the Japanese Classification of Colorectal Carcinoma 9.

### Pathology

In resected specimens (*n* = 30), according to the Japanese Classification of Colorectal Carcinoma, grade 1a tumors show denaturation and necrosis of cancer cells in approximately <1/3 of the cancer; grade 1b have denaturation and necrosis in <2/3 of cancer cells plus fusion in >1/3 of the cancer; grade 2 shows significant denaturation, necrosis, fusion, and loss in >2/3 of the cancer; and in pathological complete response (ypCR), no cancer cell is observed in both primary and regional lymph nodes 9.

In nonresected/nonoperation cases (*n* = 19), the tumor size of rectal cancer was defined by Response Evaluation Criteria in Solid Tumors 11. We classified patient response at 8 weeks after HCRT as follows: complete response (CR), that is, total disappearance of the lesions; partial response (PR), that is, 30% decrease in the sum of diameters of the lesions; stable disease (SD), that is, between 30% decrease and 20% increase; and progressive disease (PD), that is, 20% increase in the sum of diameters of the lesions or new distant metastasis. The CRPD (complete response, PD) group included patients where local tumors showed CR, but new distant metastasis appeared during HCRT.

The main endpoint of this pilot study was the pathological and clinical response after 8 weeks of treatment. Adverse effects were evaluated according to the common terminology criteria for adverse events 12.

## Results

Patient characteristics are shown in Table 1. All patients tolerated HCRT treatment without major adverse effects. One grade 3 patient had perianal dermatitis. Only two patients wanted to decrease the dose of capecitabine (complete treatment in 47/49 [95.9%] patients). No complication was observed in 64.9% patients during RF irradiation, but 29.7% suffered pain, and 2.1% had subcutaneous induration.

**Table 1:** Patients’ characteristics

	Number of cases (%)
Age (y)	
Median	62
Range	33–89
Gender	
Female	12 (24.5)
Male	37 (75.5)
Stoma	
(−)	41 (83.7)
(+)	8 (16.3)
Primary tumor	
T2	8 (16.3)
T3	36 (73.5)
T4	5 (10.2)
Regional lymph node	
N(−)	29 (59.2)
N(+)	20 (40.8)
Distant metastasis	
M0	44 (89.8)
M1	5 (10.2)
TNM stage^1^	
I	6 (12.2)
II	21 (42.9)
III	17 (34.7)
IV	5 (10.2)
Tumor differentiation	
Well differentiated	26 (53.1)
Moderately differentiated	20 (40.8)
Poorly differentiated	3 (6.1)

TNM, tumor node metastasis; CT, computed tomography; MRI, magnetic resonance imaging.

1 Tumor staging was clinical, if available, by CT and MRI.

Thirty-three patients underwent surgery 8 weeks after HCRT, mainly at the Department of General Surgical Science, Gunma University, and the Division of Surgery at Hidaka Hospital. Each resected specimen was evaluated histologically at the Department of Pathology, Gunma University. Average and median anal verge distance were 2.76 cm and 3.0 cm, respectively. Abdominoperineal resection was performed in seven (14.3%) patients. One patient could not have the main tumor resected, 5 did not have surgery because of PD, and 11 refused surgery.

Table 2 summarizes the patients’ status after 8 weeks of treatment. Local control of the tumor including CRPD was seen in 14 (18.5%) of 49 patients and in 17 (34.7%) patients with grade 3 or 2 cancer in this pilot study. Patients with less advanced tumors showed ypCR. In patients with preoperative stoma, which meant foreign matter was present in the radiation field, had an unfavorable outcome.

**Table 2:** Summary of patients’ outcomes

	Grade 3 (%)	Grade 2, 1b, 1a (%)	CR (%)	PR, SD (%)	PD (CRPD-grade 2, 1b PD-PD) (%)	Total
Total no	8 (16.3)	20 (40.8)	3 (6.1)	11 (22.4)	7 (3-2-2) (14.3)	49
Female	2 (16.7)	2 (16.7)	1 (8.3)	4 (33.3)	3 (2-0-1) (25.0)	12
Male	6 (16.2)	18 (48.6)	2 (5.4)	7 (18.9)	4 (1-2-1) (10.8)	37
Age						
–63	2 (7.7)	14 (53.8)	1 (3.8)	6 (23.1)	3 (1-1-1) (11.5)	26
64–	6 (26.1)	6 (26.1)	2 (8.7)	5 (21.7)	4 (2-1-1) (17.4)	23
Tumor differentiation						
Well differentiated	4 (15.4)	9 (34.6)	1 (3.8)	8 (30.8)	4 (2-1-1) (15.4)	26
Moderately differentiated	3 (15.0)	11 (55.0)	2 (10.0)	2 (10.0)	2 (0-1-1) (10.0)	20
Poorly differentiated	1 (33.3)	0 (0.0)	0 (0.0)	1 (33.3)	1 (1-0-0) (33.3)	3
Location of tumor						
Ra	1 (20.0)	0 (0.0)	0 (0.0)	2 (40.0)	2 (1-1-0) (40.0)	5
Rb	5 (17.2)	14 (48.3)	2 (6.9)	5 (17.2)	3 (0-1-2) (10.3)	29
RbP	2 (14.3)	6 (42.9)	0 (0.0)	4 (28.6)	2 (2-0-0) (14.3)	14
P	0 (0.0)	0 (0.0)	1 (100.0)	0 (0.0)	0 (0-0-0) (0.0)	1
Primary tumor						
T2	3 (37.5)	3 (37.5)	2 (25.0)	0 (0.0)	0 (0-0-0) (0.0)	8
T3	5 (13.9)	15 (41.7)	1 (2.8)	8 (22.2)	7 (3-2-2) (19.4)	36
T4	0 (0.0)	2 (40.0)	0 (0.0)	3 (60.0)	0 (0-0-0) (0.0)	5
Regional lymp						
N(−)	7 (24.1)	9 (31.0)	4 (13.8)	6 (20.7)	3 (1-1-1) (10.3)	29
N(+)	1 (5.0)	10 (50.0)	1 (5.0)	4 (20.0)	4 (3-1-0) (20.0)	20
Distant metast						
M0	8 (18.2)	19 (43.2)	3 (6.8)	10 (22.7)	4 (1-2-1) (9.1)	44
M1	0 (0.0)	1 (20.0)	0 (0.0)	1 (20.0)	3 (2-0-1) (60.0)	5
TNM stage^1^						
Stage 1 (T1, T2, N0)	3 (50.0)	1 (16.7)	2 (33.3)	0 (0.0)	0 (0-0-0) (0.0)	6
Stage 2 (T3, T4, N0)	4 (19.0)	8 (38.1)	1 (4.8)	6 (28.6)	2 (0-1-1) (9.5)	21
Stage 3 (N+)	1 (5.9)	10 (58.8)	0 (0.0)	4 (23.5)	2 (1-1-0) (11.8)	17
Stage 4 (M+)	0 (0.0)	1 (20.0	0 (0.0)	1 (20.0	3 (2-0-1) (60.0)	5
Stoma						
(−)	8 (19.5	18 (43.9	2 (4.9)	8 (19.5)	5 (2-1-2) (12.2)	41
(+)	0 (0.0)	2 (25.0)	1 (12.5)	3 (37.5)	2 (1-1-0) (25.0)	8
Operation						
LAR^2^	0 (0.0)	2 (66.7)	0 (0.0)	1 (33.3)	0 (0-0-0) (0.0	3
sLAR^3^	5 (45.5)	5 (45.5)	0 (0.0	0 (0.0)	1 (0-1-0) (9.1)	11
iSR^4^	1 (12.5)	6 (75.0)	0 (0.0)	1 (12.5)	0 (0-0-0) (0.0)	8
APR^5^	0 (0.0)	6 (85.7)	0 (0.0)	1 (14.3)	0 (0-0-0) (0.0)	7
Partial resection	2 (66.7)	1 (33.3)	0 (0.0)	0 (0.0)	0 (0-0-0) (0.0)	3
No resection	0 (0.0)	0 (0.0)	0 (0.0)	0 (0.0)	1 (0-1-0) (100.0)	1
No operation	0 (0.0)	0 (0.0)	3 (18.8)	8 (50.0)	5 (3-0-2) (31.3)	16

1 Tumor staging was clinical, if available, by CT and MRI.

2 Low anteriol resection.

3 Super low anteriol resection.

4 Intersphincteric resection.

5 Abdominoperineal resection.

In all, 75% specimens showed a change from T2 to T0, 69.4% showed a change from T3 to T2 or T0, and 100% showed a change from T4 to T2 or T3. In all, 10.3% specimens showed a change from N0 to N1, and 70.0% showed a change from N1 or N2 to N0. Furthermore, 9.1% showed a change from M0 to M1 and 0% showed a change from M1 to M0.

**Figure 2:**
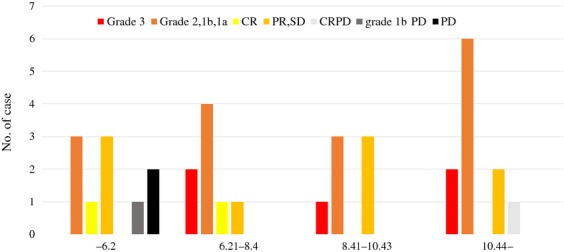
Correlation between objective response and average total accumulated irradiation output (TAIO) in 36 patients. Pathological complete response (ypCR) grade 3 and local complete response with distant progressive disease (CRPD) were seen in patients who could irradiate over 6.21 average TAIO, but not in those with 6.2 or lower average TAIO. ypCR was seen in 5 (19.2%) of 26 cases with over 6.21 TAIO. PR, partial response; SD, stable disease.

Figure 2 shows the results of the correlation between objective response and average TAIO in 36 patients. Patients showed ypCR in 5 (19.2%) of 26 cases over 6.21 TAIO. In middle and higher TAIO patients, ypCR was seen, and one case of CRPD was seen in higher output patients. In 6.2 or lower and 6.2 or higher TAIO, histological grade 3 + 2 plus clinical CR were 2 (18.2%) and 20 (52.6%), others 5 (45.5%) and 15 (39.5%), and PD 4 (36.4%) and 3 (7.9%), respectively. There was a significant difference among them (*P* = 0.028).

**Figure 3:**
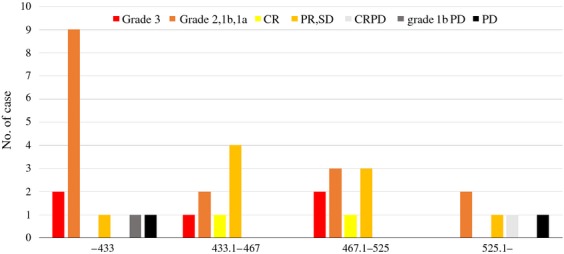
Correlation between objective response and total accumulated thermal output (TATO) in 36 patients. Local complete response with distant progressive disease (CRPD), grade 2, 1b progressive disease (PD), and PD were seen in patients with 6.2 or lower and 525.1 or higher TATO, but not in those with 433.1–525 TATO. PR, partial response; SD, stable disease.

Figure 3 shows the results of correlation between objective response and TATO in 36 patients. PD patients including CRPD and grade 2, 1b PD were observed in the lowest and highest TATO patients. There was no significant difference among them.

**Figure 4:**
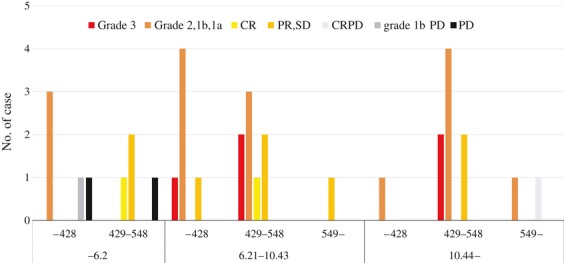
Correlation among objective response, total accumulated irradiation output (TAIO) and total accumulated thermal output (TATO) in 35 patients. In patients with 6.2 or lower TAIO, local complete response with distant progressive disease (CRPD), grade 2, 1b progressive disease (PD), and PD were seen, while in those with 10.44 or lower TAIO, one CRPD patient was present in those with 549 or higher TATO. PR, partial response; SD, stable disease.

Figure 4 shows the correlation among objective response, TAIO and TATO in 35 patients. Consequently, ypCR patients were present in the middle TAIO and TATO group, while PD patients in lowest TAIO group. In 6.2 or lower TAIO and 429 or higher TATO, ypCR plus CR was shown in only one case, while 10 (27.0%) in 6.21 or higher TAIO, and also 9 (90%) patients of total 10 ypCR plus CR patients were shown in patients with 6.21 or higher TAIO and 429 or higher TATO. From these data, we think that 6.21 or higher TAIO and 429 or higher TATO are proper output and thermal dose.

**Figure 5:**
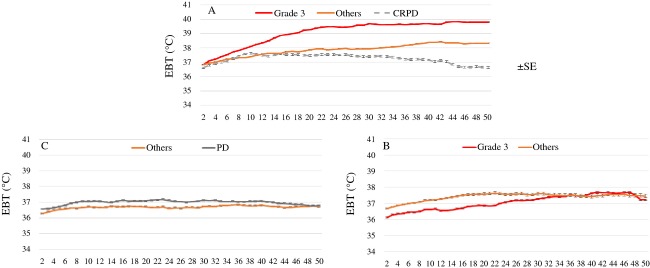
Changes in possible body temperature during 50 min irradiation based on the correlation between total accumulated irradiation output (TAIO) (A: 10.44, B: 6.21–10.43, C: 6.2) and objective response. In patients with 6.21–10.43 TAIO pathological complete response (ypCR) patients showed lower temperature (B), while in those with 10.44 or higher TAIO, ypCR patients showed higher temperature, but a CRPD patient showed lower temperature (A). Data in the figure are presented as means with standard error (SEM).

Figure 5 shows the changes of possible body temperature during 50 min irradiation according to the correlation between TAIO (Fig. 5A: 10.44, Fig. 5B: 6.21–10.43, Fig. 5C: 6.2) and objective response. Patients received over 10.44 TAIO irradiation, and their body temperature increased, their outcomes were ypCR, but not increased, their outcome was CRPD (Fig. 5A).

**Figure 6:**
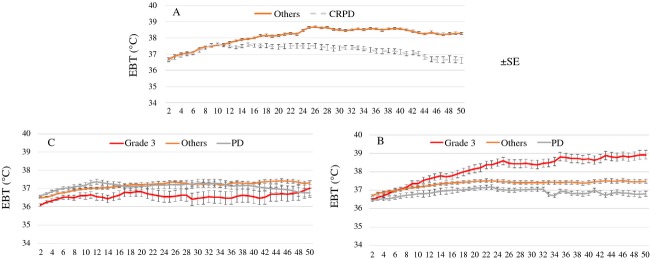
Changes in possible body temperature during 50 min irradiation according to the correlation between total accumulated thermal output (TATO) (A: 549, B: 429–548, C: 428) and objective response. In patients with 429–548 TATO, patients with pathological complete response (ypCR) showed significant increase in temperature (B), but there was no increase in those with 428 or lower TATO (C). Data in the figure are presented as means with standard error (SEM).

Figure 6 shows changes in body temperature during 50 min irradiation according to the correlation between TATO (Fig. 6A: 549, Fig. 6B: 429–548, Fig. 6C: 428) and objective response. In patients in whom body temperature increased, consequently had a good outcome (Fig. 6B), and patients in whom body temperature did not increase, still had a good outcome from this modality (Fig. 6C).

## Discussion

In the pilot study presented here, we demonstrated that 8 (24.2%) of 33 patients who underwent surgery and 16.3% of total patients experienced ypCR and local CR, 6 (37.5%) of 16 patients who were not operated showed local tumor control, 14 (28.6%) of 49 showed ypCR, CR, or CRPD, and 7 (14.3%) of 49 had clinical PD. In 11 patients who received surgery and could be 6.21 or higher TAIO and 429–548 TATO showed 4 (36.4%) ypCR patients. Moreover, 90% of ypCR plus CR patients show 6.21 or higher TAIO and 429 or higher TATO.

We concluded that patients with less advanced cancer (T2 > T3, T4) and those who could be treated with mild hyperthermia such as 6.21–10.43 TAIO and 429–548 TATO could be beneficial from RF hyperthermic treatment. But, because this study is a small sample, a conclusion has a limit.

All medical treatments are quantitatively characterized by defined parameters. It is essential to identify a parameter to quantify the effect of hyperthermia, as noted in other treatment modalities. If thermometry of one cancer cell is possible in a clinical setting, the true intracellular temperature can be measured, but this is impossible presently. Even in chemotherapy, the drug concentration in one cancer cell is impossible to determine because of tumor heterogeneity; consequently, the true intracellular drug concentration cannot be evaluated in relationship with its efficacy at present. As a result, the parameter in chemotherapy, and in radiotherapy, mg/m^2^ and Gy, respectively, is used for standardized treatment as a reference point. In clinical setting, multi-institute studies on both chemotherapy and radiotherapy have been performed using these parameters. Even the surgical treatment has no reference. For a long time, there was also no research for reference points in hyperthermic therapy.

Efforts had been made to standardize dosimetric measures of microwave/RF exposure as Watts per kilogram (W/kg) in 1981 by the National Council on Radiation Protection and Measurement 13, but there has been no new study until now.

We propose TAIO (W min^−1^ m^−2^) as a quantitative parameter of the RF hyperthermia device, which can be used to determine the exact dose (output) of a RF hyperthermia device, contraindications or indications of this treatment, conditions of optimal thermal treatment, limits of the treatment, and treatment efficacy by multi-institutional studies. As for the thermal factor, a thermometer was placed on the skin on the lateral abdominal side for simple and reproducible assessment of body temperature in patients and the new parameter of TATO (°C min^−1^ m^−2^) was defined as the hypothetical heat dosage in patients. In terms of hyperthermia therapy, the most important factor is the intratumor temperature of 42°C during treatment 14. However, due to tumor heterogeneity, even adequate intratumor temperature is not enough to kill all cancer cells with heat. Hyperthermia itself has several cellular effects that should be synergistic with radiation-induced tumor cell death. Therefore, targeting a combination of tumor-specific and DNA repair pathways will not only enhance heat-induced radiosensitization in both chemotherapy and radiotherapy but will also decrease the overall level of normal tissue toxicity during radiotherapy, which could eventually help to improve the sequential use of heat and radiation treatment to obtain a better clinical outcome 15.

The term “mild” or “physiological range hyperthermia” is found sporadically in basic research, but there are no data regarding this in a clinical setting. Mace et al. reported that antigen-specific activation of naïve CD8^+^ T cells and their differentiation into effector cells are temperature-sensitive events 16,17. These reports and our results have shown positive effects of mild/physiological hyperthermia on tumors when used along with chemoradiation. However, from our results, temperature is liable to depend on individuals. Our data suggest that certain individuals may be able to increase the set point of core temperature according to the output of RF irradiation.

On the other hand, side effects of hyperthermia are caused by large reflections at the interfaces between soft tissue and bone or air, which causes severe complications, including pain, unpleasant sensations, and burns, in 5–16% patients, and even prevents treatment from being efficacious 18–21. It is necessary to accumulate high-quality evidence by performing a multicenter study to evaluate the relationship between adequate device condition, adequate total radiation dose, and adequate chemotherapeutic regimes and dose in cancer patients together with a study of prophylaxis for complications.

Radiation therapy has become a treatment modality for many types of cancer, but is associated with long-term adverse effects. Therefore, good local tumor control without good survival has been reported in patients after radiation therapy 22. Gérard et al. 6 summarized four randomized trials of neoadjuvant treatment for rectal cancer, with ypCR ranging from 13% to 20% and grade 3 ranging from 6% to 25%. Chemotherapy consisting of oxaliplatin has also shown to increase ypCR rate and grade 3 toxicity 24,25,23. Huang et al. 26 recently summarized the results from previously published studies on the efficacy of preoperative radiotherapy plus capecitabine (825–850 mg/m^2^ twice daily, 5 days/week) in the treatment of locally advanced rectal cancer showing ypCR rates ranging from 6.7% to 31% and grade 3+ acute toxicities were noted in 5–15% of patients.

In this study, we used TomoTherapy® for treatment, which has potential advantages for rectal cancer patients, such as confirmation of the exact shape and location of a colorectal tumor, and decreased treatment-related side effects by minimizing damage to nearby healthy tissue. De Ridder et al. 27 first reported the efficacy of helical TomoTherapy® (23 fractions of 2 Gy within 5 weeks) on rectal cancer, with which only one patient developed grade 3, and this modality might decrease gastrointestinal toxicity.

The positive outcome of IRMT plus capecitabine was shown in ypCR rates ranging from 14.1% to 30.6% with grade 3 rates from 11.1% to 17.6% 28,26, but these reports did not mention PD cases. Lu et al. 29 only reported a ypCR of 20% with grade 3 at 22%, and PD in 17% of cases. Reports of controlled trials also did not mention PD. Thus, it is likely that PD cases were missed in these studies. Consequently, good local control outcomes did not correlate with good survival in patients. The timing of surgery after chemoradiation must be reconsidered in the future.

From our results and reports, the following two questions are raised:

1. Why did the body temperature of ypCR patients increase in patients who could be irradiated with a TAIO of 10.44 and higher, but not in CRPD patients?
2. Why was ypCR not seen in cases of a TAIO of 6.2 or lower?

Based on published literature, ypCR rate is less at 10–15% for rectal cancer patients treated with neoadjuvant CRT. RF therapy may offset the chemoradiation effect in these patients.

In the former patients, those with low body temperature showed ypCR. This result may be help to predict the response of patients to hyperthermia. The set point of core temperature in an individual will be a key in predicting the RF response.

As for the latter, we must have fully defined strategies such as shifting patients who received low output irradiation because of complication at present to those with more output irradiation by preventing complications.

We conclude that standardization of RF hyperthermia using an 8 MHz RF capacitive heating device can be established as a potential new treatment for rectal cancer concomitant with chemoradiation therapy.

Adding RF hyperthermia has shown good local control and low toxicity 30, and if the aforementioned problems are solved, this new combined modality provides another potential treatment in patients with rectal cancer. A randomized control study can be planned for the future using a new strategy, which consists of lower Gy and lower chemotherapeutic dose than that currently concomitant with neothermia, which can lead to good survival of patients with rectal cancer.
